# Response of the metabolic activity and taxonomic composition of bacterial communities to mosaically varying soil salinity and alkalinity

**DOI:** 10.1038/s41598-024-57430-2

**Published:** 2024-03-29

**Authors:** Márton Mucsi, Andrea K. Borsodi, Melinda Megyes, Tibor Szili-Kovács

**Affiliations:** 1 Institute for Soil Sciences, HUN-REN Centre for Agricultural Research, Herman Ottó út 15, Budapest, 1022 Hungary; 2 Doctoral School of Environmental Sciences, ELTE Eötvös Loránd University, Pázmány P. sétány 1/AC, Budapest, 1117 Hungary; 3 Department of Microbiology, ELTE Eötvös Loránd University, Pázmány P. sétány 1/C, Budapest, 1117 Hungary; 4 Institute of Aquatic Ecology, HUN-REN Centre for Ecological Research, Karolina út 29, Budapest, 1113 Hungary

**Keywords:** Biogeochemistry, Microbial communities, Environmental microbiology, Microbial ecology

## Abstract

Soil salinity and sodicity is a worldwide problem that affects the composition and activity of bacterial communities and results from elevated salt and sodium contents. Depending on the degree of environmental pressure and the combined effect of other factors, haloalkalitolerant and haloalkaliphilic bacterial communities will be selected. These bacteria play a potential role in the maintenance and restoration of salt-affected soils; however, until recently, only a limited number of studies have simultaneously studied the bacterial diversity and activity of saline–sodic soils. Soil samples were collected to analyse and compare the taxonomic composition and metabolic activity of bacteria from four distinct natural plant communities at three soil depths corresponding to a salinity‒sodicity gradient. Bacterial diversity was detected using 16S rRNA gene Illumina MiSeq amplicon sequencing. Community-level physiological profiles (CLPPs) were analysed using the MicroResp™ method. The genus-level bacterial composition and CLPPs differed significantly in soils with different alkaline vegetation. The surface soil samples also significantly differed from the intermediate and deep soil samples. The results showed that the pH, salt content, and Na^+^ content of the soils were the main edaphic factors influencing both bacterial diversity and activity. With salinity and pH, the proportion of the phylum Gemmatimonadota increased, while the proportions of Actinobacteriota and Acidobacteriota decreased.

## Introduction

More than 424 million hectares of surface soil (0–30 cm) and 833 million hectares of subsurface soil (30–100 cm) on Earth are salt-affected, with 85% of salt-affected surface soils being saline, 10% sodic and 5% saline–sodic^1^. Salt-affected soils can be divided into two main groups^2^. Saline soils contain neutral soluble salts, mainly sodium chloride and sodium sulphate but also appreciable quantities of calcium and magnesium chlorides and sulphates. The second group contains sodium salts capable of alkaline hydrolysis, mainly sodium carbonate. These soils are named ‘alkali soils’. Salt-affected soils adversely affect the growth of most crop plants. Saline and sodic landscapes are subjected to modified hydrological processes that can impact soil chemistry, carbon and nutrient cycling, and organic matter decomposition^3^. It is crucial to obtain additional knowledge about the microbiology of saline–sodic soils because the area of these soils is increasing due to anthropogenic impacts, e.g., secondary salinization^4^, as a result of improper land management and irrigation^5,6^.

Saline–sodic soils are considered harsh environments for almost all life forms and reduce microbial biomass^7–9^, soil respiration^10–13^, and the activities of various enzymes, such as urease, alkaline phosphatase, and β-glucosidase^10,14^. Increased salinity and sodicity act as environmental filters selecting tolerant and adaptive microbes and support active and diverse microbial communities^15^. In addition to salinity and sodicity, the stress effects of extreme summer heat and drought can also have a significant impact on the diversity and composition of soil microbial communities^16^. Saline–sodic habitats are ideal environments for alkaliphilic and/or halophilic bacteria, as emphasized by Horikoshi et al.^17^. Alkaliphilic microorganisms grow optimally at pH ≥ 9, while halophilic organisms grow at NaCl concentrations ≥ 1.2 M.

Most of the related studies assessing the microbiology of saline environments have focused on aquatic communities^18–20^ and sediments^21–23^, and relatively few studies have focused on understanding saline soils^24–26^. Despite the widespread occurrence of saline–sodic soils, especially in arid and semi-arid inland climate zones, the microbiome in saline–sodic soils remains largely unexplored^27^.

One of the most comprehensive studies based on 16S rRNA gene sequence analysis of soils and sediments from different environments revealed that salinity, rather than other factors, e.g., temperature, pH, or other physical and chemical factors, was the major environmental determinant of microbial community composition^28^. Another global survey of 237 locations and multiple environmental parameters revealed ultraviolet light, forest environment, soil carbon and pH to be significant and globally consistent predictors of soil bacterial diversity^29^. A previous study of saline soil from a vegetated shoreline in an arid zone showed that both soil pH and salinity were equally important in shaping the soil microbial composition^26^. In soils with broad ranges of pH values (from 4 to > 8), pH was the best predictor of microbial community structure^30,31^. In another study in which a narrow pH but broad electrical conductivity (EC) ranges were investigated, salinity was the most important factor impacting soil prokaryotic community structures^32^. Studies of microbial communities in saline and sodic soils usually focused almost exclusively on the surface soil layer, and only one study investigated the subsurface (15–30 cm) adjacent to the surface soil^25^. Groundwater depletion, caused mainly by increased water use, as found in Kiskunság National Park, located in Hungary, has resulted in a gradual decrease in salt concentration and pH in the surface soil, leading to rapid changes in soil development and vegetation composition^33^. Therefore, we selected this area as a model area for following the changes in soil salinity and sodicity under different vegetation types and at different soil depths.

The aims of this research were to (1) reveal the bacterial community composition along the salinity and sodicity gradient represented by four distinct alkaline plant communities in surface, intermediate and deep soils; (2) characterize the community-level physiological profiles along the salinity and sodicity gradient from the surface to subsurface soil; and (3) identify the environmental (edaphic) factors responsible for the differentiation of bacterial diversity and metabolic activity.

## Results

### Soil physical and chemical properties

The soils (AL, AP, and AA) could be characterized generally as saline–sodic, except for those at the AF site, which had a neutral pH and low EC in the 0–10 cm layer and a slightly alkaline pH and low EC in the 10–30 cm layer (Table 1). The salt pioneer sward (AL) site had the highest pH and highest EC in the surface soil, while the other sampling sites had the maximum values in either the intermediate (AP site) or the deep (AA and AF sites) soils. The soil texture varied among clay, clay loam, silty clay loam, silt loam, and loam. The soil organic carbon content was the highest in the upper 10-cm layer and gradually decreased with depth at all four sites. The soil organic C and soil total N varied in the opposite direction with the soil pH and EC among the sites. As expected, multiple strong and significant positive correlations were found between most of the soil properties (Supplementary Fig. 1), especially pH_H2O_, pH_KCl_, EC and Na, which were closely correlated with each other. These parameters were negatively correlated with SOC and TN, which confounded the identification. These soil properties were the main drivers of the catabolic activity profiles and microbial community structure.

**Table 1:** Most important physical and chemical soil properties of the Apaj soil samples from four distinct natural plant communities at three soil depths.

Site	Depth	pH_H2O_	pH_KCl_	EC (mS cm^−1^)	SOC (%)	TN (g kg^−1^)	CaCO_3_ (%)	NO_3_-N (mg kg^−1^)	AL-K_2_O (mg kg^−1^)	AL-P_2_O_5_ (mg kg^−1^)	AL-Na (mg kg^−1^)	Mn (mg kg^−1^)
AL	a	10.4 ± 0.2	9.6 ± 0.3	5.11 ± 1.49	0.37 ± 0.10	0.39 ± 0.09	24.6 ± 7.4	4.4 ± 1.8	193 ± 5	35 ± 16	4224 ± 714	32.14 ± 12.88
	b	10.5 ± 0.0	9.8 ± 0.1	4.76 ± 0.23	0.17 ± 0.03	0.28 ± 0.04	42.6 ± 5.0	1.6 ± 0.5	151 ± 35	16 ± 6.8	4191 ± 177	12 ± 5.33
	c	10.5 ± 0.0	9.7 ± 0.0	3.17 ± 0.47	0.10 ± 0.01	0.20 ± 0.02	34.3 ± 2.00	0.9 ± 0.0	78 ± 4	19 ± 11	2951 ± 316	12.25 ± 1.57
AP	a	9.7 ± 0.1	9.00 ± 0.1	3.05 ± 0.19	1.02 ± 0.23	1.32 ± 0.36	22.2 ± 1.2	4.3 ± 1.1	175 ± 21	88 ± 15	3571 ± 144	48.62 ± 3.93
	b	10.1 ± 0.0	9.3 ± 0.1	3.45 ± 0.3	0.32 ± 0.03	0.50 ± 0.06	32.9 ± 2.9	0.3 ± 0.5	182 ± 12	20 ± 2.6	3459 ± 77	23.78 ± 1.82
	c	10.3 ± 0.0	9.00 ± 0.1	1.8 ± 0.2	0.14 ± 0.01	0.25 ± 0.00	35.8 ± 4.3	0.3 ± 0.5	123 ± 11	12 ± 4.0	2143 ± 48	16.11 ± 2.26
AA	a	9.4 ± 0.1	8.00 ± 0.0	1.24 ± 0.14	0.79 ± 0.02	1.13 ± 0.04	15.1 ± 2.6	6.9 ± 1.1	118 ± 9	45 ± 8.3	1787 ± 326	24.13 ± 3.62
	b	10.3 ± 0.1	9.1 ± 0.3	3.15 ± 0.79	0.25 ± 0.05	0.35 ± 0.04	26.3 ± 9.8	0.3 ± 0.5	180 ± 8	20 ± 4.3	3599 ± 553	19.04 ± 5.62
	c	10.5 ± 0.0	9.5 ± 0.1	3.04 ± 0.28	0.12 ± 0.02	0.20 ± 0.02	36.6 ± 4.7	0.0 ± 0.0	97 ± 26	15 ± 13	3035 ± 116	10.75 ± 0.93
AF	a	7.7 ± 0.1	7.3 ± 0.0	0.29 ± 0.01	1.76 ± 0.11	2.07 ± 0.17	14.2 ± 1.8	1.7 ± 0.1	257 ± 53	113 ± 6.9	84 ± 10	49.48 ± 6.85
	b	8.2 ± 0.1	7.4 ± 0.0	0.26 ± 0.02	1.13 ± 0.10	1.30 ± 0.09	15.8 ± 2.6	0.2 ± 0.4	167 ± 38	86 ± 15	153 ± 56	38.54 ± 4.97
	c	9.4 ± 0.3	7.9 ± 0.2	0.42 ± 0.19	0.40 ± 0.03	0.44 ± 0.02	38.6 ± 1.1	0.4 ± 0.6	108 ± 13	20 ± 2.1	968 ± 538	11.99 ± 0.48

*EC* electrical conductivity (mS cm^−1^), *AL* salt pioneer sward, *AP Puccinellia* sward, *AA Artemisia* alkali steppe, *AF Achillea* alkali steppe.

Soil depths: a—0–10 cm; b—10–30 cm; c—30–60 cm; 1, 2, 3: replicates; n = 3.

### Soil catabolic profiles (basal and substrate-induced respiration rates)

The basal respiration rates differed among the sampling sites and soil depths (Supplementary Fig. 2), with higher respiration rates occurring in the surface soils of the *Artemisia* and *Achillea* alkali steppes (AA and AF) than in that of the salt pioneer and *Puccinellia* sward (AL and AP). In the intermediate and deep soil samples, significantly lower basal respiration rates were measured; these rates were similar at each site. In line with this, the surface soil samples showed higher respiration rates for all substrates than did the subsurface samples. In the soils from the AP and AL sites, the variations in respiration rates were low for most substrates. Respiration rates for all substrates were lower in the subsurface samples. The AL and AP samples had substantially lower respiration rates for all the substrates than did the AF and AA samples (Supplementary Fig. 3).

The CLPPs differed significantly among sites (F = 13.299, *p* = 0.001) and depths (F = 16.520, *p* = 0.001) according to PERMANOVA. Pairwise comparisons revealed that AF significantly differed from AP and AL (*p* < 0.001 for both) and that AA differed from AL (*p* = 0.023), while the most closely associated sites in terms of CLPP were AA and AF (*p* = 0.066). Pairwise comparison by depth showed that intermediate and deep soil samples were not different, while they significantly differed from the surface soil and the other samples (*p*_adj_ < 0.05); however, the PERMANOVA results could be affected by the non-homogeneous dispersion of the data with depth (F = 6.404, *p* = 0.004), which is explained by the greater variability in catabolic profiles in the surface soil. Nevertheless, based on the PERMANOVA, the separation of the catabolic profiles was obvious.

**Figure 1:**
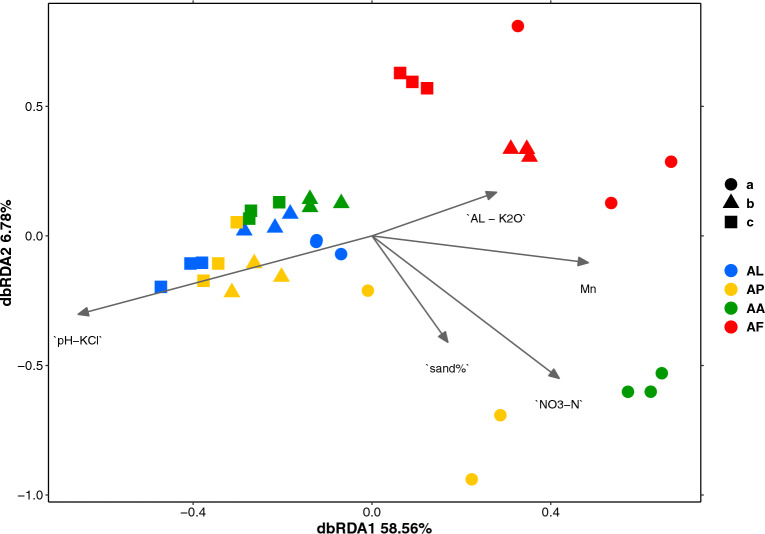
DbRDA ordination plot including the soil variables that best explained the catabolic activity profiles of the soils measured by MicroResp™. pH-KCl: soil pH in 1 N KCl extract; AL-K_2_O: ammonium-lactate extractable potassium; Mn: manganese; NO_3_-N: soil nitrate-N; sand%: soil sand content. The abbreviations of the sampling sites are given in Table 1.

Distance-based redundancy analysis (dbRDA) resulted in a model in which pH_KCl_, available K and Mn, NO_3_^−^-N and sand% were the most important soil variables influencing the catabolic activity profiles (Fig. 1). This model was found to be highly significant (F = 16.83, *p* = 0.001) when considering the 5 factors in the ANOVA (Type III): pH_KCl_ (F = 34.78, *p* = 0.001), NO_3_^−^-N (F = 13.79, *p* = 0.001), Mn (F = 3.778, *p* = 0.030), sand% (F = 2.742, *p* = 0.047), and available K (F = 2.200, *p* = 0.106) (Supplementary Table S1). The overall variance explained by the constrained axes was 73.72% while that explained by the unconstrained axes was 26.28%. The importance of the dbRDA1 component was 58.56%, while the first two constrained components (dbRDA1 + dbRDA2) accounted for 65.3% of the cumulative variance, which indicated that 48.1% of the total variance of the model could be explained by the first two axes.

The SIMPER test was used to compare standardized substrate-induced respiration rates (SIRs) and determine the relative importance of substrates at sites with different vegetation types in the surface soil samples. The SIRs of 6–10 different substrates were responsible for at least 60% of the total contributions from the 23 substrates (Supplementary Table S2), but a few of them were significantly different according to pairwise comparisons. Considering the significance levels, the AL and AP samples appeared most closely related, e.g., they were less different from each other, while the largest difference was found between the AL-AA and AL-AF samples. According to the SIR values, ascorbic acid, alanine, lysine, and arabinose were the main substrates responsible for site separation, while malic acid, succinic acid, citric acid and asparagine were the main substrates responsible for the separation of the AA and AF samples.

### Diversity and taxonomic composition of the bacterial communities

The 16S rRNA gene amplicon sequencing results provided insight into the taxonomic composition of the studied bacterial communities. After the processing of the raw data, a total of 562,625 bacterial 16S rRNA gene sequences were assigned to 9,051 OTUs. The number of sequences per sample ranged between 11,242 and 61,923. The bacterial rarefaction curves indicated that the sequencing depth was adequate (Supplementary Fig. 4).

**Figure 2:**
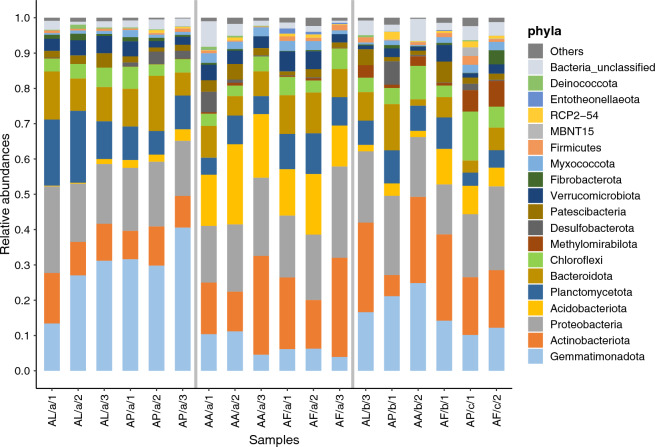
Distribution of bacterial phyla with relative abundances of 1% in at least one sample from the four distinct natural plant communities at three soil depths, based on 16S rRNA gene amplicon sequencing. The abbreviations of the sampling sites are given in Table 1.

Altogether, 42 bacterial phyla and candidate divisions were identified from the samples, of which 18 phyla (Fig. 2) were present in one of the samples with a relative abundance higher than 1%. The sequences of these taxa together accounted for more than 93% of the relative abundances in each sample. At the phylum level, distinct differences were observed between the surface soil samples designated AL/a—AP/a and AA/a—AF/a. The salt pioneer (AL) and *Puccinellia* sward (AP) samples were clearly dominated by representatives of the phylum Gemmatimonadota (mean abundance for AL/a of 23.9% and AP/a of 33.9% compared to AA/a of 8.7% and AF/a of 5.4%). The opposite trend was observed for the representatives of the phylum Actinobacteriota, whose relative abundance was approximately twice as high on average in the *Artemisia* and *Achillea* alkali steppe (AA/a—AF/a) surface soil samples than in the salt pioneer and *Puccinellia* sward (AL/a—AP/a) surface soil samples. The proportion of sequences belonging to the phylum Acidobacteriota was also the highest in the *Artemisia* and *Achillea* alkali steppe (AA/a—AF/a) surface soil samples (on average 18.4% and 13.9%, respectively). Representatives of the phylum Planctomycetota showed similar abundances among the surface soil samples only in the salt pioneer sward (AL), with an average of 16.6%. The relative abundance of sequences belonging to the phylum Proteobacteria, however, did not vary significantly by vegetation type or soil depth (ranging from 14.1% for AP/c to 23.7% for AF/c). Among the more uncommon taxa, representatives of the phylum Methylomirabilota were more abundant in the deep soil samples (AP/c and AF/c).

**Figure 3:**
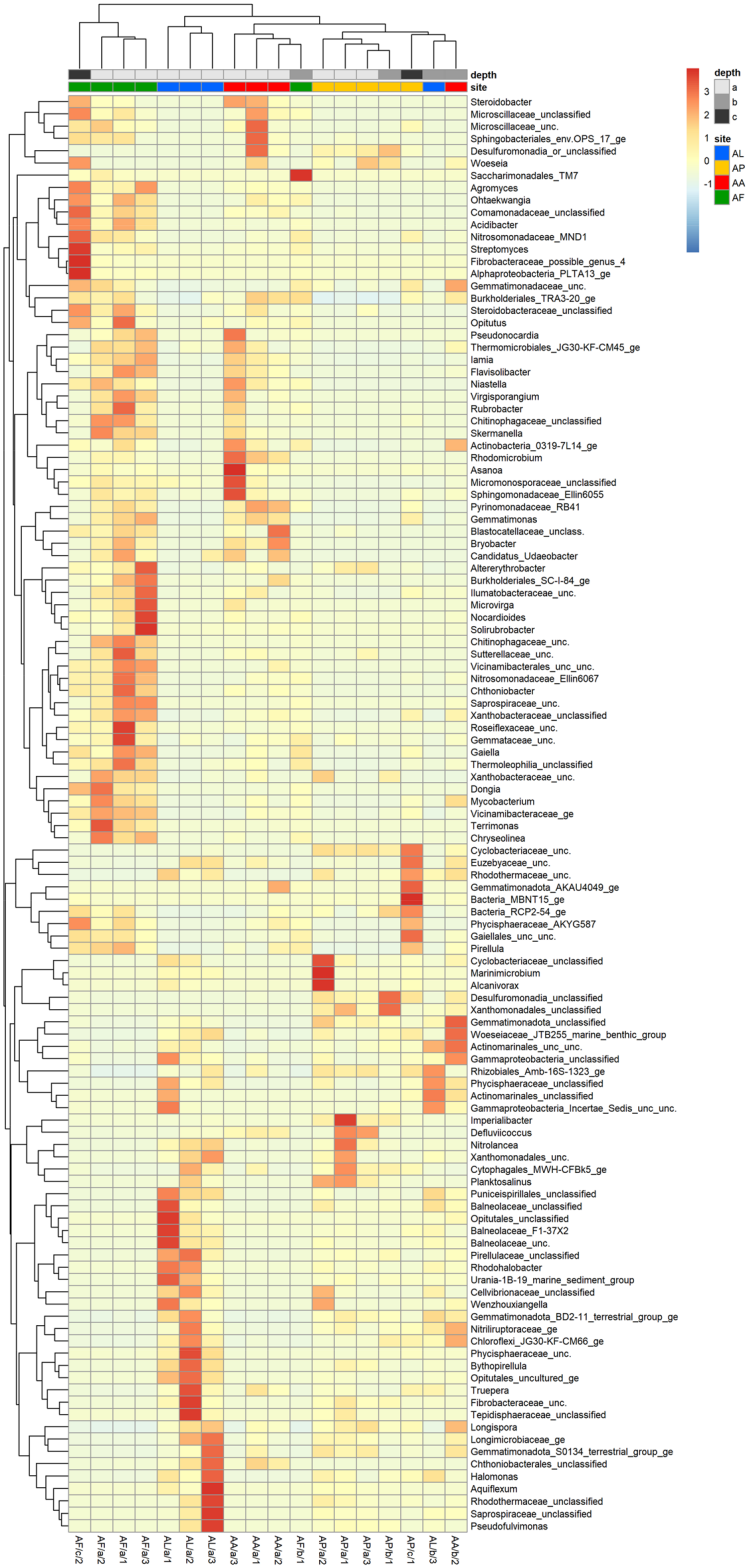
Similarity heatmap of the genus-level bacterial OTUs from the saline and alkaline soil samples of four distinct natural plant communities at three soil depths based on 16S rRNA gene amplicon sequencing. The abbreviations of the sampling sites are given in Table 1.

Hierarchical cluster analysis based on bacterial OTUs identified at the genus level resulted in the separation of two distinct groups (Fig. 3). The most different group contained only *Achillea* alkali steppe (AF) soil samples. The other large group was separated into three additional clusters, two of which mainly comprised surface salt pioneer and *Artemisia* alkali steppe (AL and AA) soil samples, while the third cluster included all the *Puccinellia* sward (AP) and other intermediate and deep soil samples. Most of the identified and sample-specific bacterial taxa (e.g., *Streptomyces*, *Nocardioides*, *Solirubrobacter*, *Mycobacterium*, *Altererythrobacter*, *Microvirga*, *Chthoniobacter*, *Dongia*, *Terrimonas*, and *Chryseolinea*) were found in the AF soil samples with the highest vegetation cover. In contrast, mainly unclassified (e.g., *Balneolaceae*, *Optitutales*, *Phycisphaeraceae*, *Fibrobacteraceae*, *Tepidisphaeraceae*, *Rhodothermaceae*, and *Saprospiraceae*) bacterial taxa were characteristic of the most extreme AL soil samples, in addition to a few known halophilic and/or marine genera (e.g., *Rhodohalobacter*, *Halomonas*, *Aquiflexum*).

### Bacterial richness and alpha diversity metrics and correlation with soil properties

Good’s coverage showed that the sequencing could cover almost all the bacterial diversity (Table 2). The number of sequences showed no clear tendencies, and the OTU and taxon numbers and richness and diversity estimates were generally greater for the *Achillea* alkali steppe (AF) samples and lower for the *Puccinellia* and salt pioneer sward (AP and AL) samples. There were positive correlations between the bacterial richness and alpha diversity indices and the SOC, total N, ammonium-N, available phosphorus and potassium and silt content, while negative correlations were found with the EC, pH, and Na^+^ concentration (Table 3).

**Table 2:** Number of sequences, OTUs, coverage, and diversity indices of the saline and alkaline soil samples from four different natural plant communities at three soil depths based on 16S rRNA gene metagenomic data.

Sample ID	No. of high-quality sequences		No. Sobs	Good’s coverage	Species richness		Diversity indices	
	Total	Sub-sampled			Chao 1	ACE	Shannon	Inv. Simpson
AL/a/1	33,522	11,242	558.8 ± 7.1	0.99	640.9 ± 19.4	642.4 ± 14.9	5.1	72.0 ± 1.3
AL/a/2	47,228	11,242	781.7 ± 10.1	0.98	971.0 ± 34.6	971.5 ± 26.2	5.5	99.7 ± 2.1
AL/a/3	43,202	11,242	827.7 ± 10.9	0.98	1057.7 ± 39.1	1057.5 ± 30.5	5.6	127.4 ± 2.4
AP/a/1	25,957	11,242	745.3 ± 8.3	0.98	868.0 ± 24.0	889.4 ± 19.7	5.3	74.6 ± 1.3
AP/a/2	28,213	11,242	581.0 ± 7.0	0.99	664.9 ± 19.1	672.4 ± 15.6	5.1	55.6 ± 1.2
AP/a/3	20,834	11,242	485.5 ± 5.4	0.99	539.2 ± 13.9	556.9 ± 12.6	4.5	30.6 ± 0.5
AA/a/1	31,760	11,242	740.2 ± 8.4	0.98	860.3 ± 24.0	873.6 ± 19.6	5.1	59.3 ± 1.0
AA/a/2	15,408	11,242	815.5 ± 4.2	0.99	851.1 ± 8.4	880.6 ± 9.1	5.5	93.9 ± 1.2
AA/a/3	15,517	11,242	1279.3 ± 6.0	0.98	1354.7 ± 11.6	1438.2 ± 14.7	5.7	72.2 ± 1.1
AF/a/1	35,527	11,242	1738.5 ± 15.8	0.95	2242.1 ± 53.2	2356.6 ± 48.6	6.5	288.5 ± 6.0
AF/a/2	26,993	11,242	1556.6 ± 12.9	0.96	1881.7 ± 38.1	1979.1 ± 35.8	6.3	226.5 ± 4.3
AF/a/3	27,441	11,242	1065.6 ± 8.4	0.98	1176.1 ± 20.2	1191.4 ± 16.9	6.0	183.6 ± 3.1
AL/b/3	29,722	11,242	597.0 ± 6.8	0.99	667.6 ± 17.3	670.2 ± 13.7	4.9	47.3 ± 0.9
AP/b/1	15,618	11,242	609.4 ± 4.2	0.99	643.9 ± 8.8	666.4 ± 9.0	5.0	59.4 ± 0.7
AA/b/2	61,923	11,242	635.4 ± 7.4	0.99	710.3 ± 18.8	705.5 ± 14.0	5.0	55.8 ± 1.1
AF/b/1	11,242	11,242	1050.0 ± 0.0	1.00	1052.0 ± 0.0	1062.9 ± 0.0	5.9	133.5 ± 0.0
AP/c/1	53,380	11,242	690.9 ± 5.9	0.99	734.4 ± 13.4	725.0 ± 9.0	5.5	99.0 ± 2.0
AF/c/2	39,138	11,242	889.8 ± 8.7	0.99	990.5 ± 21.4	994.5 ± 17.1	5.6	94.7 ± 1.9

The abbreviations of the sampling sites are given in Table 1.

**Table 3:** Correlations (Pearson’s r) between species richness and the α diversity of soil bacteria and soil edaphic factors in the saline–alkaline soil samples.

	pH_H2O_		pH_KCl_		EC		AL_Na		SOC	
	r	*P*	r	*P*	r	*P*	r	*P*	r	*P*
Sobs	− 0.75	0.0003	− 0.68	0.0021	− 0.55	0.0168	− 0.68	0.0021	0.69	0.0016
Chao 1	− 0.75	0.0003	− 0.68	0.002	− 0.56	0.0166	− 0.68	0.002	0.69	0.0015
ACE	− 0.76	0.0003	− 0.68	0.002	− 0.56	0.0164	− 0.68	0.002	0.69	0.0015
Shannon	− 0.77	0.0002	− 0.73	0.0005	− 0.63	0.0047	− 0.78	0.0001	0.61	0.0074
Invsimps	− 0.78	0.0001	− 0.65	0.0034	− 0.53	0.0233	− 0.7	0.0012	0.71	0.0011

	AL_K_2_O		AL_P_2_O_5_		Total_N		NH_4__N		Silt	
	r	*P*	r	*P*	r	*P*	r	*P*	r	*P*
Sobs	0.48	0.0457	0.47	0.0486	0.62	0.0063	0.81	< 0.0001	0.47	0.0514
Chao 1	0.48	0.0452	0.47	0.0475	0.62	0.0061	0.81	< 0.0001	0.47	0.0506
ACE	0.48	0.0446	0.48	0.0462	0.62	0.0058	0.81	< 0.0001	0.47	0.0495
Shannon	0.36	0.146	0.43	0.0721	0.53	0.0231	0.68	0.0019	0.45	0.0626
Invsimps	0.57	0.013	0.57	0.0141	0.61	0.0072	0.73	0.0007	0.57	0.0137

*Sobs* actual observed richness, *ACE* ACE index of species richness, *Chao 1* Chao 1 index of species richness, *Shannon* Shannon diversity index, *Invsimps* inverse Simpson index (n = 18).

### Environmental factors affecting site variation

The bacterial communities characterized by NGS at the genus level were significantly different by sampling site (F = 4.286, *p* = 0.001) according to the PERMANOVA results. Pairwise comparisons revealed the largest significant differences between AF—AL and AF—AP (*p* = 0.009 for both), followed by AA—AF (*p* = 0.019) and AA—AP (*p* = 0.040); a marginally significant difference between AA—AL (*p* = 0.053); and a close association between AL and AP (*p* = 0.068); however, after correction for multiple sampling, differences were significant only for the AF—AL and AF—AP comparisons. The insufficient amount and non-homogenous dispersion of the data by soil depth did not allow for comparisons by soil depth using PERMANOVA; however, it is obvious that the abundance was strongly affected by depth.

**Figure 4:**
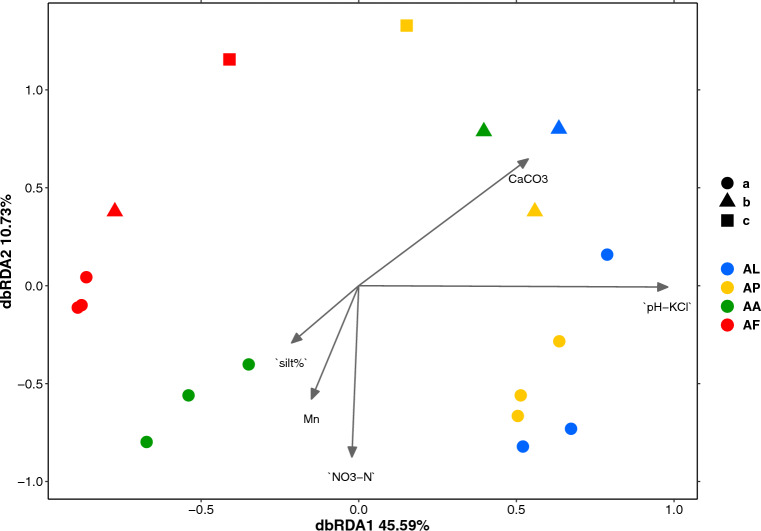
DbRDA ordination plot including the soil variables that best explained the community data obtained via genus-level NGS of the soil samples from the four vegetation sites at three soil depths. CaCO3: lime content of the soil; pH-KCl: soil pH in 1 N KCl extract; NO_3_-N: soil nitrate-N; Mn: manganese; silt%: soil silt content. The abbreviations of the sampling sites are given in Table 1.

To assess the soil variables that best explained the community structure, dbRDA was performed using a stepwise selection model after selecting non-correlated soil variables. The best explanatory model of the dbRDA (Fig. 4) was significant (F = 5.94, *p* = 0.001), which included 5 soil variables in the ANOVA test (Type III): pH_KCl_ (F = 8.22, *p* = 0.001), NO_3_^−^-N (2.33, *p* = 0.047), silt% (F = 3.271, *p* = 0.014), Mn (F = 3.356, *p* = 0.016), and CaCO_3_ (F = 2.336, *p* = 0.057) (Supplementary Table S3). The overall variance explained by the constrained axes was 71.23% while that explained by the unconstrained axes was 28.77%. The importance of the dbRDA1 component was 64.01%, while the first two constrained components (dbRDA1 + dbRDA2) accounted for 79.07% of the cumulative variance, which indicated that 56.32% of the total variance of the model could be explained by the first two axes.

The ordination diagram clearly shows that, along the first axis, the salt pioneer (AL) and *Puccinellia* sward (AP) samples separated from the *Artemisia* and *Achillea* alkali steppe (AF and AA) samples. The two latter samples are separated along the second axis. The AL and AP samples were more similar to each other than were the AF and AA samples. The latter also exhibited greater separation by soil depth (a and b) than did the AL and AP samples. However, the samples showed no clear separation according to sampling depth, partly because fewer samples from the deeper soil layers could be used for sequencing.

## Discussion

Salinization and sodification of soils can cause many problems worldwide both for agriculture and ecology^34,35^. Naturally occurring salt-affected soils, however, are also important for nature conservation purposes, providing unique habitats for unique flora^33,36^. The Apaj alkaline steppe is a nature conservation area within Kiskunság National Park, where the vegetation is well described, and the micromosaic-like alkali vegetation has many protected species, e.g., *Tripolium pannonicum* ssp. *Pannonicum*^36^.

This study is a direct continuation of the research that focused on soil microbial interactions, plant communities and soil properties during extreme dry and wet events, but only in rhizosphere soils from the same sites. In agreement with Borsodi et al.^16^, the present study confirmed that soil bacterial catabolic activity and taxonomic composition, characterized by both MicroResp™ and 16S rRNA gene sequencing of the surface soil samples, corresponded well to vegetation type and mostly formed distinct groups in the ordinations (Fig. 1). An important difference compared to the previously observed group separation was that the ordination of MicroResp™ CLPPs clearly distinguished salt pioneer (AL) and *Puccinellia* sward (AP) surface soil samples, in contrast to the metagenomic analysis. The edaphic properties of the surface AL and AP soil samples were very similar, both having pH > 9.5 and high salinity. This may have favoured colonisation by very similar halophilic and alkalophilic bacterial species, irrespective of the vegetation type. At the AP site, however, the slightly lower but still high salt concentration allowed the development of richer vegetation. At the time of sampling, in October, the AP site was covered by active and relatively dense vegetation, whereas the AL site was covered by only patchy vegetation. The amount of carbon compounds easily available to bacteria was therefore greater at the AP site than at the AL site, which may have compensated for the adverse effects of salinity^37^. In addition, the DNA-based approach could include not only active but also inactive (endospores or dormant) community members^38^. Furthermore, many bacteria can tolerate a wide range of pH and EC conditions, which allows them to grow in soils with vastly different pH and salinity^39^. All this may explain why the taxonomic composition of the bacterial communities was very similar based on 16S rRNA gene amplicon sequencing, whereas the abundance of metabolically active bacteria at the time of sampling differed significantly between sampling sites. In non-saline soils, this difference could also be explained by the activity of fungi; however, as fungi are more sensitive to salinity and high pH, the fungal/bacterial ratio in saline soil is typically very low^40^.

The overall inhibitory effects of salinity and sodicity on the soil basal respiration rate and microbial biomass are well known^9,14,40,41^. The results of the present study are in line with these effects, as shown by the comparison of basal respiration rates in the surface soil samples. Studies on the effect of soil organic matter (SOM) on microbial respiration in saline and sodic soils have reported contradictory results, as reviewed by Singh^41^. In the present study, no significant difference was found between the basal respiration rates of the AL and AP surface soil samples, despite the latter containing almost three times more SOM. This finding agrees with that of Mavi et al.^42^, who reported that while microbial respiration decreased with salinity, it was not affected by sodicity, even though the dissolved organic carbon concentration increased in more sodic soils.

Direct gradient analysis revealed that the main environmental variable influencing the soil catabolic activity profile was soil pH. This seemingly contradicts the findings of previous studies of the area, as Borsodi et al.^16^ reported that the main soil property influencing the catabolic activity profiles of surface soil samples was EC. This apparent contradiction can, however, be resolved when the connection between EC and pH is considered. In saline–sodic soils, salts that cause salinity are also sources of alkalinity. Indeed, soil pH and EC had a strong correlation (r_Pearson_ = 0.9), which means that they explained most of the same variance in the microbiological data. In their robust study on datasets of soils from areas with different land uses, Moscatelli et al.^43^ also found that MicroResp™ depended more on soil pH than on organic carbon. They also found that in alkaline soils, which they defined as having a pH > 7.4, contrary to the common definition of pH > 8.5^44^, MicroResp™ failed to differentiate between samples. In the present study, all the samples had a pH above 7.4, even in the surface soil layers; moreover, except for the F/a samples, every sample was alkaline according to the common definition. Nonetheless, we found that MicroResp™ was able to differentiate between samples from different vegetation covers and even between surface and subsurface samples, although not between samples from the intermediate and deep soil layers, which could be explained by very low catabolic activities for all substrates in the subsurface soils. Direct gradient analysis of the Illumina-based 16S rRNA gene sequence data showed that soil pH was the most important environmental variable shaping the composition of soil bacteria in the investigated alkali–saline soils, similar to the MicroResp™ results. The AF + AA soil samples were clearly separated from the AP + AL surface soil samples. The distinction between AF and AA was also strong, but the surface samples of AP and AL could not be significantly (*p* < 0.05) separated.

Concerning the taxonomic composition of the bacterial communities, the results of the present study showed that the number of identified phyla did not differ significantly among the samples; only the number of OTUs was greater at the AF site and lower at the AL and AP sites. The same was true for the diversity indices, which is in line with the findings of Borsodi et al.^16^. According to the meta-analysis of Ma and Gong^45^, Proteobacteria, Actinobacteriota, Firmicutes, Acidobacteriota, Bacteroidota and Chloroflexi were the predominant phyla in saline soils. Other studies have shown that Proteobacteria, Bacteroidota, Actinobacteriota and Firmicutes were the dominant phyla in saline–sodic soils^46–48^. The presence of Proteobacteria was considered “salinity-related”^48,49^. In our study, the phylum Proteobacteria was also abundant, but there was no significant difference in its relative abundance among the soils with different pH values and salt contents. Several studies have shown that Bacteroidota is also a dominant phylum in saline–alkali soils^50–52^, but this was not confirmed in our study. Members of the phylum Bacteroidota have wide EC tolerances; moreover, some members are extremely halophilic and are more abundant under extreme salinity conditions^52,53^. Other studies have reported that Bacteroidota and Gemmatimonadetes are important participants in biogeochemical transformations in soils under saline conditions^45,54^. The relative abundance values of some dominant phyla, however, differed significantly according to vegetation type and/or soil depth. One notable change was observed in the phylum Gemmatimonadetes, whose abundance was greatest in the AL and AP surface soils, which had the highest salinity and pH, while strong decreases in abundance were found with decreasing pH and conductivity and increasing vegetation cover. This variation in abundance is in good agreement with the previously observed high proportion of the phylum Gemmatimonadetes in arid soils and its adaptability to low-moisture environments^55^. In a study of a natural salinity–sodicity gradient in the Songnen Plain of Northeast China, Guan et al.^27^ also reported that Gemmatimonadetes exhibited the strongest preference for high salinity–sodicity. A high proportion of Gemmatimonadetes was also found in soils with elevated pH and salinity in other studies^48,52,56–58^. Interestingly, in the present study, in the subsurface soils of the densely vegetated *Artemisia* and *Achillea* alkali steppes (AA and AF), where high pH and conductivity values were also measured, the proportion of the phylum Gemmatimonadetes was twice as high as that in the surface soils. Gemmatimonadetes are known to occur in diverse habitats, such as freshwater, wastewater, sediment, soil and rhizosphere^59^. Most of them cannot be cultivated; currently, six species are described in this phylum, two of which are capable of anoxygenic photosynthesis, while the others are chemoorganoheterotrophs^60,61^. Gemmatimonadota are the eighth most abundant bacterial phylum in soils, accounting for approximately 1–2% of soil bacteria worldwide^62^. Gemmatimonadota are suggested to be adapted to dry environments because they occur in high relative proportions in semiarid and arid soils and deserts^58,63^. These bacteria prefer neutral pH but can also dominate alkaline^64^ and highly saline soils and account for nearly 17% of all the bacterial sequence reads^27^. A possible explanation for the enrichment of soil communities with Gemmatimonadetes in addition to their tolerance to drying could be that their phototrophic strains provide energy for survival^65^ at high salinities, which was also observed in the case of Cyanobacteria^27^.

The proportion of sequences belonging to the phylum Acidobacteriota and Actinobacteriota was greater in the *Artemisia* and *Achillea* alkali steppe (AA/a—AF/a) surface soils than in the more alkaline AP and AL soils. Actinobacteriota and Acidobacteriota have been depicted as common inhabitants of all soils. The relative abundance of Actinobacteriota can be greater^56,66^ or lower than that in this study. Yang et al.^52^ reported that Actinobacteriota were more abundant in saline–-alkaline soils than in low-salinity soils. The genus *Nitriliruptor* within the phylum Actinobacteriota was found to be the most abundant genus in saline–alkaline pastures^56^ and in arid saline soil in Xinjiang, Northwest China^47^. OTUs belonging to the haloalkaliphilic Nitriliruptoraceae were also abundant in the AL and AP soil samples, although the relative abundance of the phylum Actinobacteriota was lower than that in the AA and AF samples.

Similar to Gemmatimonadota, the phylum Acidobacteriota is widespread in soils, but only a few cultivated species have been described. Many Acidobacteriota are acidophilic, but they can occur in different soil environments, and their different subdivisions can be either positively or negatively correlated with soil pH^67^. Most studies agreeing with this established a negative correlation between the relative abundance of Acidobacteriota and soil salinity^32,52,58,66^. Acidobacteriota are known as oligotrophic bacteria, and they can also exhibit sensitive responses to fertilizer use^68^.

Changes in the bacterial community composition along saline–sodic gradients could be the result of many factors covarying with salinity and sodicity. The major driving factors identified as responsible for the alterations in the bacterial communities in the soil were pH, EC and soil organic matter^27,45,52^. In addition, nutrient level^26^, soil moisture^16,56^ and sulphate^48^ can also be important factors affecting community composition.

A meta-analysis showed that the global microbial diversity and composition in saline soils are more affected by salinity than by other extreme soil factors, such as pH or organic carbon^45^. It is important to note that soil salinity and pH commonly have a collinear relationship in saline–alkaline soils^26,52^, as in this study. Both the salinity and high pH resulted from the increased level of Na^+^; therefore, their effects are difficult to separate.

Although it was not possible to determine the statistical significance of the difference between the bacterial community structures of the surface and subsurface samples due to the insufficient number of samples available for evaluation, the subsurface soil samples were clearly separated from the surface soil samples in both the ordination of catabolic activity profiles and that of bacterial community structures. This finding contradicts that of Xie et al.^25^, who reported no difference between surface and subsurface bacterial communities in an extremely saline area. The relative abundance of the phylum Verrucomicrobiota was the highest in the subsurface soils due to their oligotrophic strategies in the work of Li et al.^57^, while in our study, the phylum Methylomirabilota was more abundant in the deep soil samples. This is probably due to the increased salt translocation from the surface to the deeper soils, as supported by our soil chemistry data, because of the upwards flow events of the salt-rich water have been reduced by the decline in groundwater levels. Future studies are needed because similar edaphic properties in different biogeographical regions combined with different land use types may result in various microbial compositions in alkali–saline soils.

To our knowledge, no studies have attempted to find direct connections between 16S rRNA gene metagenomic data and catabolic activity profiles, although studies that have reported both^69,70^ or linked substrate-induced respiration rates to diversity measures^71^ exist. In the present study, both the MicroResp™ and genus-level ordinations were more similar to the ordination of soil properties than to each other. This difference might be a result of functional redundancy in the bacterial communities, as a carbon source can be metabolized by many different bacterial taxa, and different groups can substitute each other across varying samples. However, the similarity between the two ordinations suggested that there is a link between bacterial community composition and function. This study provides a description of the bacterial communities in soils with different levels of salinity and sodicity, which could provide a starting point for restoring these fragile habitats^72^. Most likely, the most significant agricultural application is the isolation of haloalkalitolerant bacteria from saline–alkaline environments with plant growth-promoting properties to support crop plant health and resistance against soil salinity^73–75^.

## Conclusion

This study revealed that the bacterial community and community-level physiological profile (CLPP) significantly differed in soils along a salinity‒sodic gradient. The surface soil and subsurface soil (> 10 cm) also significantly differed. In catabolic responses after different substrate additions, ascorbic acid, alanine, lysine, and arabinose were the main substrates responsible for site separation in the CLPP. Soil bacterial alpha diversity responded to soil pH and salinity, with significantly lower values of the Shannon and Chao 1 indices recorded in plant communities with increased pH, salinity and Na^+^. The phylum-level bacterial community composition was comparable among the sites; however, the representatives of Gemmatimonadota were more abundant in the most saline-alkali soils, whereas the abundances of Actinobacteria and Acidobacteria decreased. Methylomirabilota were more abundant in the deep soil layers than in the surface layers. Separation of the bacterial composition at the genus level and in the CLPP across the four vegetation sites was influenced mainly by the soil pH, which was also collinear with EC and Na^+^. Presumably, the elevated concentration of Na^+^ accounts for the improvements in both the pH and EC in this saline-alkali area.

## Materials and methods

### Study site and sampling

**Figure 5:**
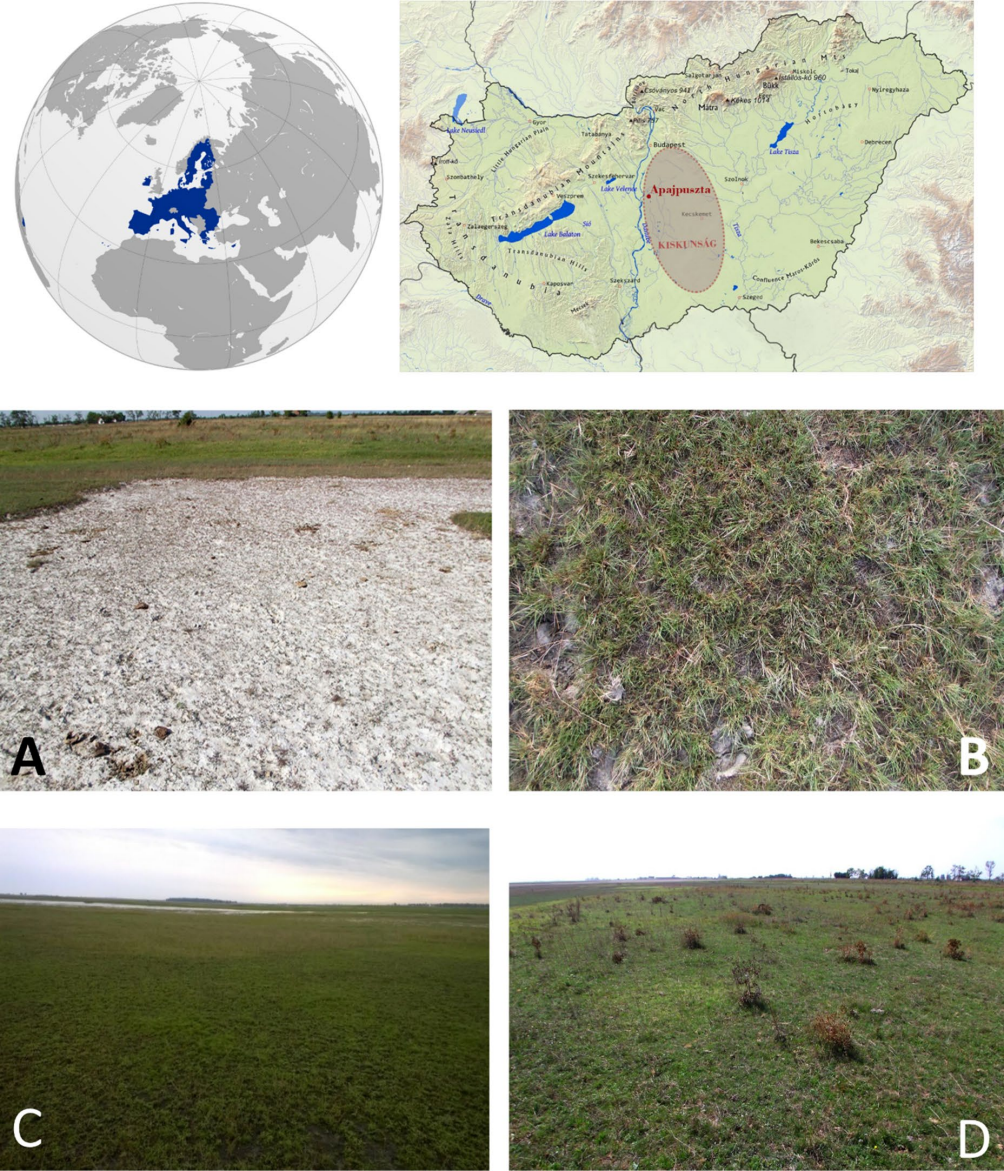
Location and vegetation types of the sampling sites. (**A**) Salt pioneer sward (AL, *Lepidio crassifolii-Camphorosma annuae*); (**B**) Puccinellia sward (AP, *Lepidio crassifolii-Puccinellietum limosae*); (**C**) Artemisia alkali steppe (AA, *Artemisio santonici-Festucetum pseudovinae*); (**D**) Achillea alkali steppe (AF, *Achilleo-Festucetum pseudovinae*).

The sampling area was Kiskunság National Park near the village of Apaj, Hungary (Fig. 5), between 47° 05′ 11.60′′ N, 19° 05′ 54.40′′ E and 47° 05′ 08.50′′ N, 19° 06′ 07.10′′ E at elevations between 93 and 94 m above sea level. This area is characterized by a mosaic of different levels of soil sodicity and salinity. Small relief differences can result in significant differences in salt concentrations in surface soils, which leads to patchy, mosaic-like structures^76^. The distance of the ground level from the groundwater level is well indicated by the varying alkali vegetation types, as different plant associations can tolerate different degrees of salinity and sodicity^36^. Sampling sites were therefore selected according to typical plant associations^77^: AL—Salt pioneer sward (*Lepidio crassifolii-Camphorosma annuae*), AP—*Puccinellia* sward (*Lepidio crassifolii-Puccinellietum limosae*), AA—*Artemisia* alkali steppe (*Artemisio santonici-Festucetum pseudovinae*), and AF—*Achillea* alkali steppe (*Achilleo-Festucetum pseudovinae*). The grassland vegetation-covered soils are not cultivated, and their maintenance involves mainly cattle grazing.

In the sampling area, the climate is temperate, with a 10 °C annual mean temperature (minimum of − 2 °C in January and maximum of + 21 °C in July); the average annual sum of precipitation is 527 mm; and the mean annual potential evaporation is 900 mm. The average depth of the groundwater level is 1.6 m, ranging from a minimum of 0.6 m and a maximum of 2.3 m^78^.

Soil samples were collected with a corer (5 cm diam.) on 10 October 2016 from four sites representing the main plant associations in the area. At each site, three plots (1 m × 1 m) were established, and multiple cores were taken from each plot to a depth of approximately 60 cm. Then, the cores were cut into three sections according to depth: 0–10 cm (a), surface; 10–30 cm (b), intermediate; and 30–60 cm (c), deep. Approximately 1 kg of soil sample from the same plot and depth was mixed in disposable polyethylene (PE) bags, resulting in three replicates per site and depth. The samples were subsequently transported to the laboratory on the same day. After thorough mixing, the samples were divided into three parts: approximately 10 g was placed in sterile microtubes for subsequent DNA isolation, approximately 300 g was stored in PE bags at 4 °C for physiological tests (catabolic activity), and approximately 500 g was air-dried for soil physical and chemical analyses.

### Determination of soil physical and chemical properties

The soil physical and chemical properties were measured using the standard Hungarian methodology^79,80^. The gravimetric water content was determined by oven- drying at 105 °C for 24 h. The soil pH was measured in a 1:2.5 soil-to-water suspension (pH_H2O_) and in a soil-to-KCl solution (1 mol) suspension (pH_KCl_). The soil particle size distribution was determined via the sedimentation method. The soil organic carbon content was measured via dichromate oxidation. The lime content (CaCO_3_) was determined using a calcimeter, and the electrical conductivity was measured using an EC meter in a water-saturated soil paste (ECe). The concentrations of some macro- and microelements were measured via inductively coupled plasma atomic emission spectrometry (ICP‒AES), and the soluble nutrient content was determined with ammonium-lactate (AL) for K_2_O, P_2_O_5_ and Na or with EDTA/KCl extractant for Mn.

### Catabolic activity of the soil samples

Community-level physiological profiles (CLPPs) were measured by the MicroResp™ method^81^ using 23 different substrates and distilled water as a control. Soil samples were prepared by sieving through a 2-mm-mesh screen and manually removing visible roots and stones, setting the soil water holding capacity to 45%, then transferring into 96-well deep-well plates (one sample per plate). The plates were covered with Parafilm M and put in desiccators for 3 days at 25 °C for pre-incubation. Then, 25 µl of substrate solutions were added to each plate in four-well replicates. The following substrates were used: D-galactose (Gal), trehalose (Tre), L-arabinose (Ara), D-glucose (Glc), and D-fructose (Fru) at 80 g/L, citric acid (Cit), DL-malic acid (Mal), Na-succinate (Suc), L-alanine (Ala), and L-lysine (Lys) at 40 g/L, L-glutamine (Gln) at 20 g/L, L-arginine (Arg), and 3.4-dihydroxybenzoic acid (Dhb), and L-glutamic acid (Glu) at 12 g/L, myo-inositol (Ino), D-xylose (Xyl), D-mannitol (Mat), D-mannose (Man), D-sorbitol (Sor), and L-rhamnose (Rha) at 80 g/L, L-asparagine-monohydrate (Asn) at 20 g/L, and D-gluconic-acid-potassium (Gla) and L-ascorbic acid (Asa) at 40 g/L. The pH of the substrate solutions was adjusted to neutral (pH = 7). The plates were left uncovered for 20 min to avoid the detection of CO_2_ from potential abiotic processes. Then, the plates were covered with detector plates containing purified Oxoid agar gel with a cresol red indicator and incubated for 6 h at 25 °C. CO_2_ development rates (μg CO_2_-C g soil^−1^ h^−1^) were calculated as the colour change in the detector plates measured with a photometer (Anthos 2010; Biochrom, Cambridge, UK) at a 570 nm wavelength before and after incubation. CO_2_ calculations were performed according to the manufacturer’s instructions. Basal respiration rates were calculated from wells filled with distilled water as controls.

### Bacterial diversity analysis by 16S rRNA next-generation sequencing (NGS)

Environmental DNA was isolated from approximately 0.5 g of soil from each sample using a PowerSoil Kit (MO BIO Laboratories, Inc., Carlsbad, USA) according to the manufacturer’s instructions. All the samples from the surface (0–10 cm) soil provided adequate DNA concentrations. Furthermore, only one sample per site from the intermediate soils, and only one sample from the deep soils supplied a sufficient amount of DNA. This is not unusual in extreme soils; e.g., Xie et al.^25^ also used the same extraction kit and found that 7 out of 15 saline soil samples provided insufficient DNA for high-throughput sequencing.

PCR amplification was performed in triplicate using the B341F (5′-CCT ACG GGN GGC WGC AG-3′) and B805R (5′-GAC TAC NVG GGT ATC TAA TCC-3′) universal bacterial primers^82^ with tags on the 5′ ends targeting the V3-V4 region of the 16S rRNA gene. The PCR mixture contained 1 μl of template DNA, 0.2 μl of Phusion Hot Start II High-Fidelity DNA Polymerase (2 U/μL), 4 μl of 5 × Phusion HF Buffer (Thermo Scientific), 4 μl of dNTP mixture (10 μM), 0.4 μl of BSA (20 mg/ml, Thermo Scientific) and 0.3 μl of bacterial primers (40 μM) in 9.8 μl of nuclease-free water. The thermal conditions for PCR were as follows: initial denaturation at 98 °C for 3 min, 25 cycles of denaturation (95 °C for 10 s), annealing (55 °C for 30 s) and extension (72 °C for 30 s) and a final extension step at 72 °C for 5 min. PCR amplicons were pooled, and their concentrations were normalized using a Qubit dsDNA HS Assay Kit (Thermo Fisher Scientific). The barcoded libraries were sequenced on an Illumina MiSeq platform (Illumina, Inc., San Diego, USA) at the Genomics Core, Research Technology Support Facility (Michigan State University, Trowbridge, USA) using a MiSeq Reagent Kit v2 (500 cycles, 2 × 250-bp paired ends). The raw sequence data were deposited under the NCBI BioProject ID PRJNA987868.

Analysis of the resulting sequence reads was performed by Mothur v1.38.1^83^ based on the MiSeq SOP (www.mothur.org/wiki/MiSeq_SOP)^84^ with the following exceptions: ‘‘deltaq’’ adjusted to 10 in the ‘‘make.contigs’’command. UCHIME was used for chimera detection^85^. Singleton reads were removed from the dataset as described by Kunin et al.^86^. Taxonomic assignments were made by using a minimum bootstrap confidence score of 80%, which was calculated after 1000 iterations and based on the ARB-SILVA SSU Ref NR 132 database^87^. The latter database was also used as a reference in the former sequence alignment step. Operational taxonomic units (OTUs) were generated by applying a 0.15 cut-off in Mothur’s ‘‘dist.seqs’’ command and subsequently using 97% 16S rRNA gene sequence similarity, corresponding to the prokaryotic species-level threshold of^88^. Diversity indices and richness estimators were calculated with Mothur.

OTUs classified other than as Bacteria were excluded from the analyses. Richness and diversity indices (e.g., ACE, Chao 1, inverse Simpson) were calculated from the datasets rarefied to the same sequence depth (*n* = 11,242) using Mothur.

### Statistical analysis

Statistical analysis was performed using R 4.1.2^89^ with the vegan package 2.5-7^90^. Pearson’s correlations were calculated using the *rcorr* function of the Hmisc package 4.4-1^91^. Hierarchical cluster analysis was performed after selecting the most abundant genera using the pheatmap (Pretty heatmap) package 1.0.12^92^. PERMANOVA^93^ with the *adonis* function was used to assess the significant differences among sites and soil depths in the case of soil properties, NGS data and catabolic activity profiles. When significant differences were found, pairwise PERMANOVA was applied using the pairwiseAdonis package 0.0.1^94^ with Bonferroni correction. To explore which substrate respiration rates were responsible for distinguishing soils, the SIMPER test was used^95^. Distance-based redundancy analysis (dbRDA) was performed to relate the microbial community measures to the soil physical and chemical properties, and the MicroResp™ and NGS data at the genus level were analysed separately^96^. Distance-based redundancy analysis (dbRDA) based on Bray‒Curtis distances was performed using the *capscale* function of the vegan package; this method is a constrained ordination method that does not require linear or unimodal relationships between explanatory and response variables^96^. The relative abundance data were Hellinger-transformed before calculating the Bray‒Curtis distances. Because the soil data exhibited high multicollinearity, the soil variables were pre-selected based on their explanatory power. This was done by running dbRDA with each soil variable separately, which gave how much of the microbial data could be linked to each soil variable if considered alone. Soil variables with low explanatory power (defined as explaining less than 5% of the overall variance in the microbial data) were excluded from the dbRDA models. The best fitting RDA model was selected based on the Akaike information criterion (AIC) with bidirectional stepwise model selection using the *ordistep* function of the vegan package. The resulting models were tested for multicollinearity again, and the significance of the models was calculated. Where the soil variables still showed multicollinearity, ecologically meaningful choices were made to exclude or include the variables in question.

### Supplementary Information

Supplementary Information 1.

Supplementary Information 2.

## Data Availability

The original sequences of this study have been deposited in the NCBI Sequence Read Archive (SRA) under accession numbers SRR25033134-SRR25033151 under BioProject PRJNA987868. The data for the soil chemical properties and MicroResp™ are available in Supplementary Data S1.
