# Supplementary material for: Response of the metabolic activity and taxonomic composition of bacterial communities to mosaically varying soil salinity and alkalinity
Source: Sci Rep. 2024 Mar 29;14:7460. doi: 10.1038/s41598-024-57430-2 (PMC10980690; doi:10.1038/s41598-024-57430-2)
Supplement: Supplementary file 1 — Supplementary Information 1. [file 41598_2024_57430_MOESM1_ESM.docx]

**Supplementary materials**

**Journal name:** Scientific Reports

**Manuscript Title: Response of the metabolic activity and taxonomic composition of bacterial communities to mosaically varying soil salinity and alkalinity**

**Authors:** Márton Mucsi^1,2^, Andrea K. Borsodi^3^, Melinda Megyes^2,3^, Tibor Szili-Kovács^1^*

**Affiliations:**

^1^Institute for Soil Sciences, HUN-REN Centre for Agricultural Research, Herman Ottó út 15, H-1022 Budapest, Hungary

^2^Doctoral School of Environmental Sciences, ELTE Eötvös Loránd University, Pázmány P. sétány 1/AC, H-1117 Budapest, Hungary

^3^Department of Microbiology, ELTE Eötvös Loránd University, Pázmány P. sétány 1/C, H-1117 Budapest, Hungary

*https://orcid.org/0000-0002-8354-0656

**Correspondence:**

**Tibor Szili-Kovács**

Institute for Soil Sciences, HUN-REN Centre for Agricultural Research,

Herman Ottó út 15, H-1022 Budapest, Hungary

Tel: +36-30-961-7452

E-mail: [szili-kovacs.tibor@atk.hun-ren.hu](mailto:szili-kovacs.tibor@atk.hu)

**Andrea K. Borsodi**

Department of Microbiology, ELTE Eötvös Loránd University, Pázmány P. sétány 1/C, H-1117 Budapest, Hungary

Tel: +36-1-372-2500/8793

E-mail: borsodi.andrea@ttk.elte.hu

**
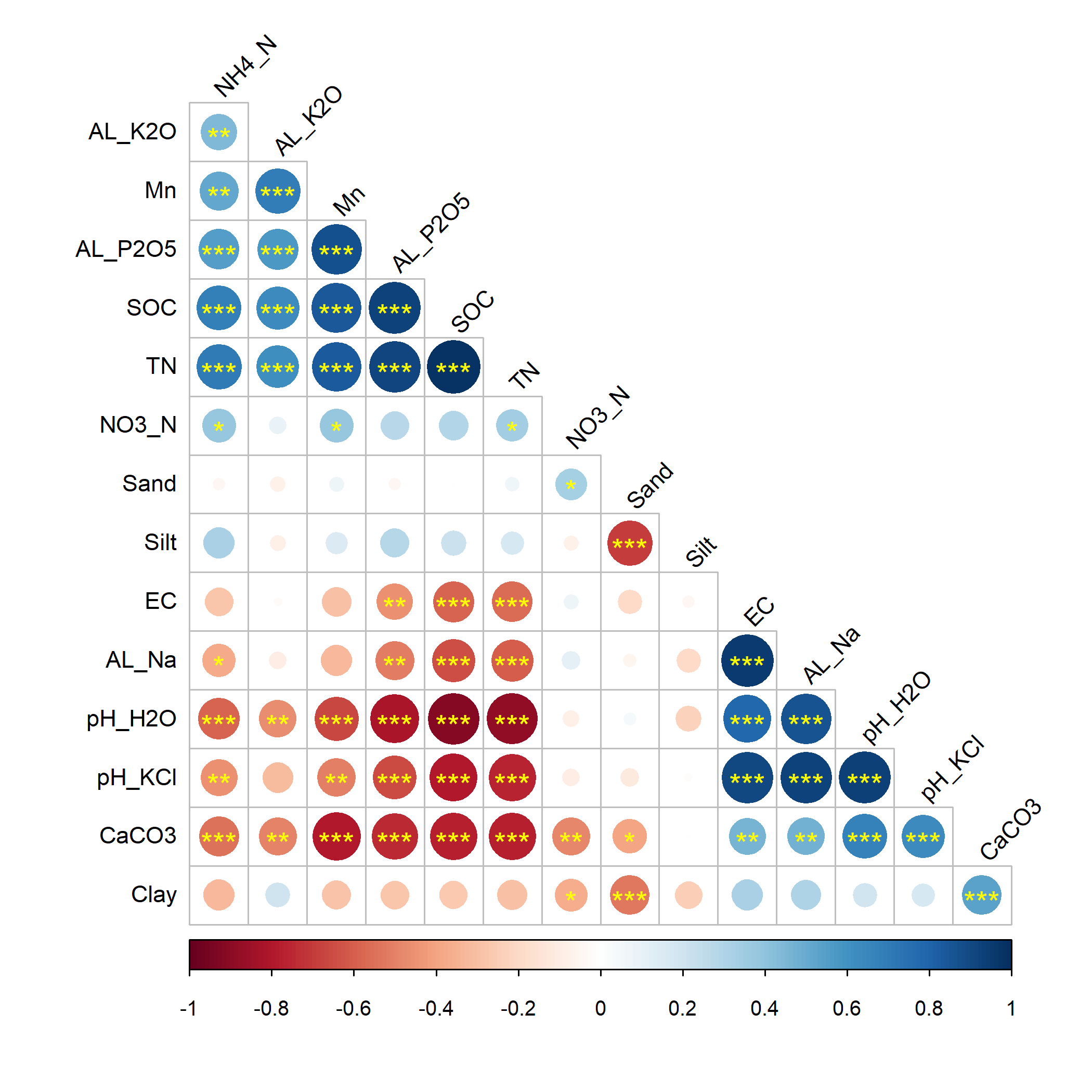
**

**Supplementary Figure** **1.** Correlations between soil physical and chemical properties. Size and color of the circles denote Pearson’s r value. Significant correlations are indicated by asterisks (* P < 0.05; ** P < 0.01; *** P < 0.001).

**
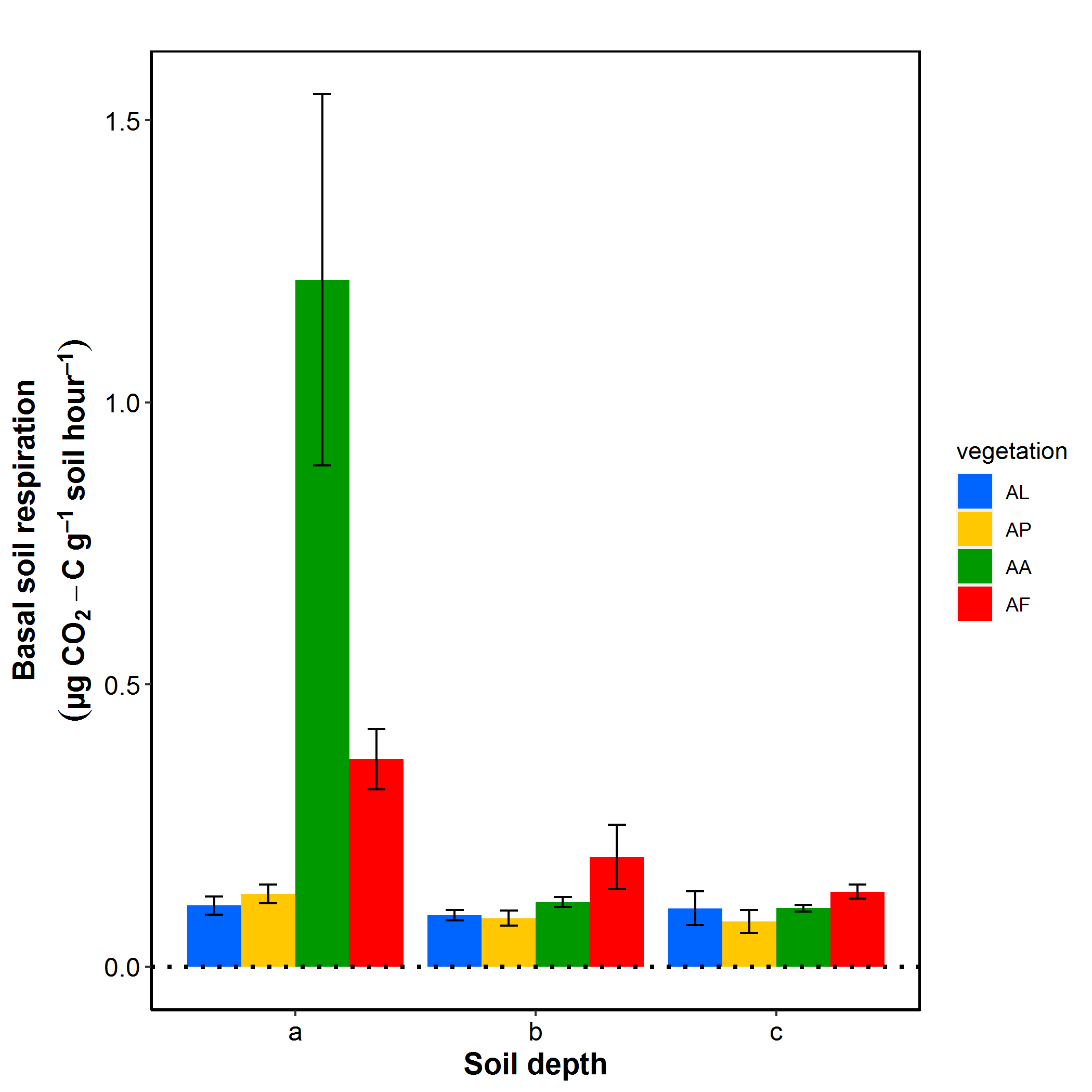
**

**Supplementary Figure 2**. Mean basal soil respiration rates (+/- standard deviation) by MicroResp (without substrate addition) originated from different alkali vegetation sites (AL, AP, AA, AF) in 3 soil depth, a:0-10cm, b:10-30cm, c:30-60cm (The abbreviations of the sampling sites are given in Table 1.)

**
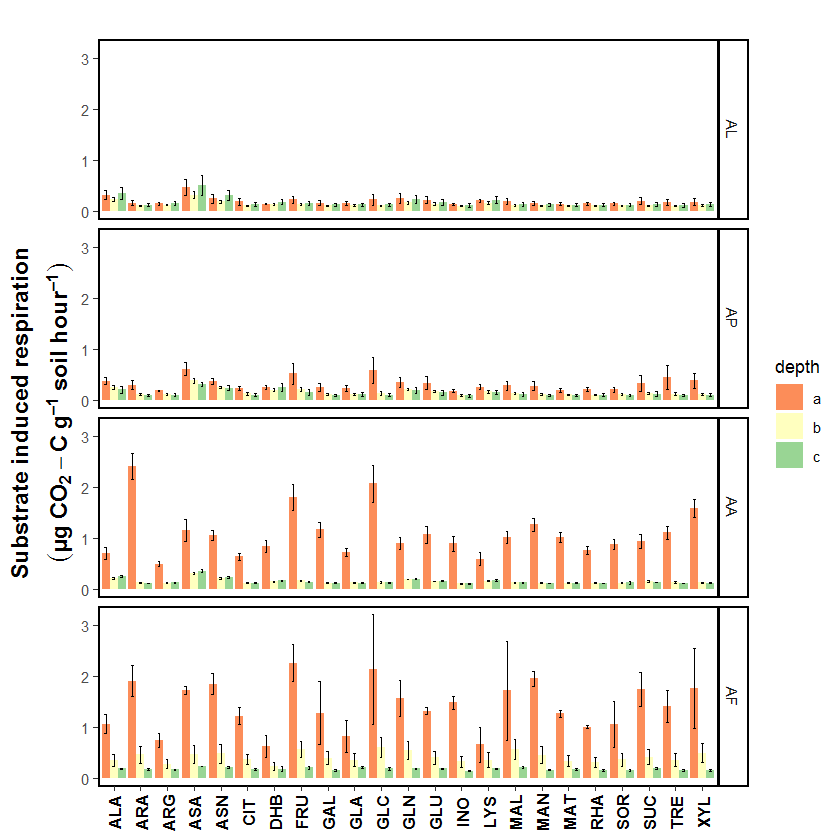
**

**Supplementary Figure 3.** Mean substrate induced respiration rates (+/- standard deviation) for the 23 substrates measured by MicroResp originated from 4 vegetation sites (AL, AP, AA, AF) from 3 soil depth, a:0-10cm, b:10-30cm, c:30-60cm. (The abbreviations of the sampling sites are given in Table 1.)


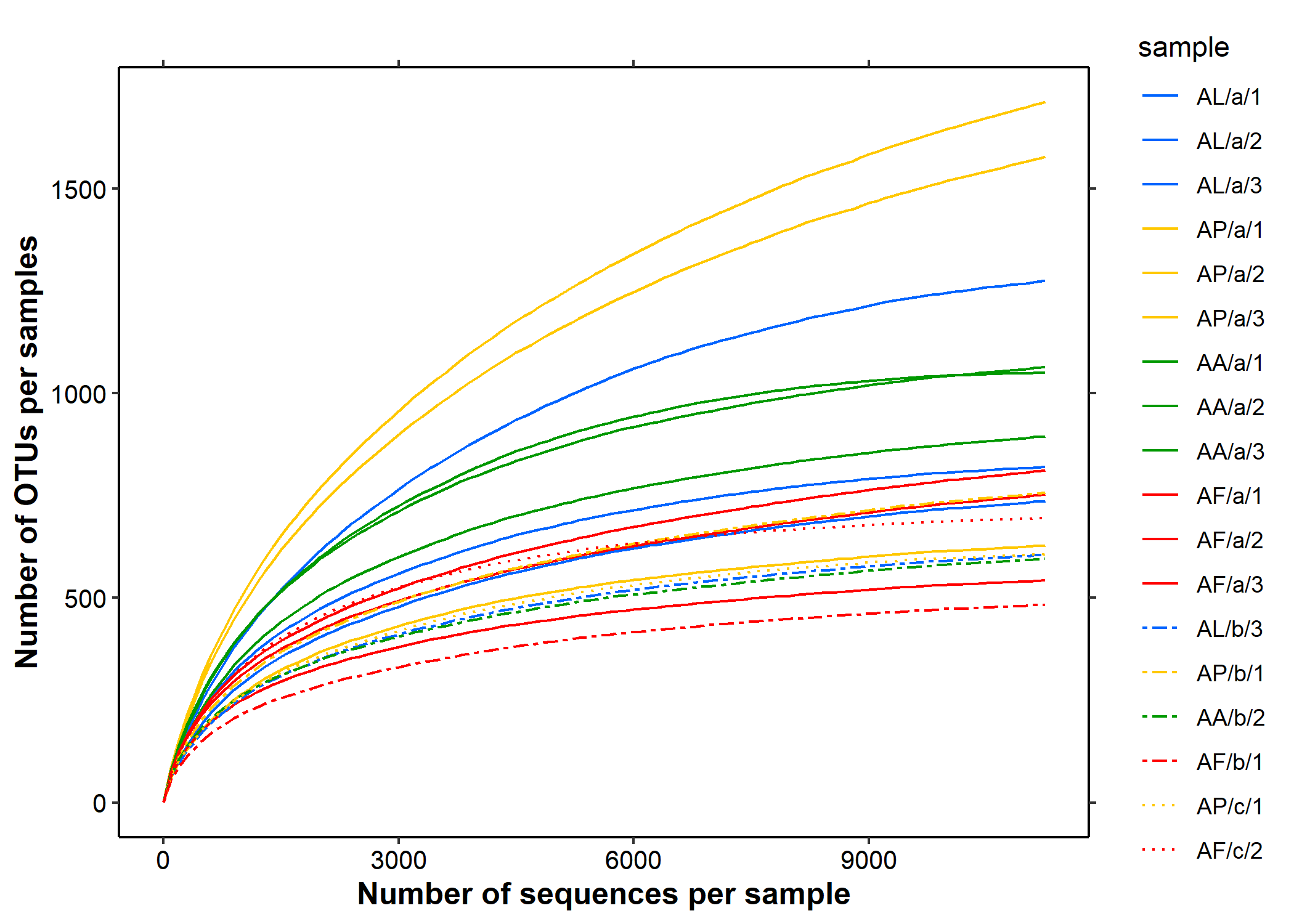


**Supplementary Figure 4.** Rarefaction curves of the 18 samples showing the bacterial OTUs in the Apaj soil samples from four different natural plant communities at three soil depths.

**Table S1.** Analysis of variance of the microResp data, model based on dbRDA reduced model with 5 factors, with permutation.

|  |  | Type I test |  |  | Type III test |  |  |
| --- | --- | --- | --- | --- | --- | --- | --- |
|  | Df | Sum of Sqs | F | *p* | Sum of Sqs | F | *p* |
| Model | 5 | 0.2794 | 16.83 | 0.001 |  |  | 0.001 |
| pH_KCl_ | 1 | 0.1749 | 52.67 | 0.001 | 0.1155 | 34.78 | 0.001 |
| NO_3_^–^-N | 1 | 0.0667 | 20.08 | 0.001 | 0.0458 | 13.79 | 0.001 |
| Mn | 1 | 0.0213 | 6.42 | 0.003 | 0.0125 | 3.78 | 0.030 |
| sand% | 1 | 0.0092 | 2.78 | 0.061 | 0.0091 | 2.74 | 0.047 |
| AK | 1 | 0.0073 | 2.20 | 0.092 | 0.0073 | 2.20 | 0.106 |
| Residual | 30 | 0.0996 |  |  | 0.0996 |  |  |

AK: available potassium. Analyses all terms separately in a sequential (Type I) test, terms added sequentially (first to last). Type III tests analyse the marginal effects when each term is eliminated from the model containing all other terms.

**Table S2.** SIMPER test results comparing the “a” (0-10 cm) layer of the four sites to identify which substrate induced respiration rates are important in discriminating among pairs of sites. The order of substrates reflects their power of discriminating between the two sites, only those substrates are presented which cumulative contribution reached 60%.

| Contrast: AL_AP | |  |  |  |  |  |  |
| --- | --- | --- | --- | --- | --- | --- | --- |
|  | average | sd | ratio | ava | avb | cumsum | P |
| GLC | 0.0153 | 0.0082 | 1.868 | 1.103 | 1.824 | 0.145 | 0.041 |
| TRE | 0.0103 | 0.0073 | 1.406 | 0.871 | 1.355 | 0.242 | 0.04 |
| FRU | 0.0099 | 0.0039 | 2.549 | 1.158 | 1.625 | 0.337 | 0.18 |
| ASA | 0.0090 | 0.0043 | 2.096 | 2.409 | 1.985 | 0.422 | 0.979 |
| ALA | 0.0088 | 0.0020 | 4.389 | 1.642 | 1.229 | 0.505 | 0.848 |
| XYL | 0.0058 | 0.0040 | 1.446 | 0.928 | 1.197 | 0.560 | 0.865 |
| LYS | 0.0048 | 0.0030 | 1.612 | 1.071 | 0.844 | 0.606 | 0.944 |

| Contrast: AL_AF | | |  |  |  |  |  |
| --- | --- | --- | --- | --- | --- | --- | --- |
|  | average | sd | ratio | ava | avb | cumsum | P |
| ASA | 0.0242 | 0.0039 | 6.194 | 2.409 | 1.269 | 0.127 | 0.012 |
| ALA | 0.0182 | 0.0031 | 5.933 | 1.642 | 0.783 | 0.223 | 0.007 |
| MAN | 0.0139 | 0.0043 | 3.252 | 0.785 | 1.440 | 0.296 | 0.001 |
| LYS | 0.0129 | 0.0040 | 3.201 | 1.071 | 0.465 | 0.363 | 0.002 |
| ARA | 0.0125 | 0.0037 | 3.359 | 0.810 | 1.399 | 0.429 | 0.702 |
| GLC | 0.0115 | 0.0077 | 1.492 | 1.103 | 1.499 | 0.489 | 0.404 |
| FRU | 0.0115 | 0.0091 | 1.263 | 1.158 | 1.695 | 0.549 | 0.042 |
| MAL | 0.0103 | 0.0033 | 3.110 | 0.978 | 1.202 | 0.604 | 0.039 |

| Contrast: AL_AA | | |  |  |  |  |  |
| --- | --- | --- | --- | --- | --- | --- | --- |
|  | average | sd | ratio | ava | avb | cumsum | p |
| ARA | 0.0300 | 0.0013 | 22.629 | 0.810 | 2.201 | 0.150 | 0.001 |
| ASA | 0.0293 | 0.0020 | 14.385 | 2.409 | 1.051 | 0.297 | 0.001 |
| ALA | 0.0217 | 0.0008 | 28.369 | 1.642 | 0.638 | 0.405 | 0.001 |
| GLC | 0.0168 | 0.0047 | 3.550 | 1.103 | 1.882 | 0.489 | 0.023 |
| LYS | 0.0115 | 0.0027 | 4.242 | 1.071 | 0.538 | 0.547 | 0.009 |
| XYL | 0.0113 | 0.0018 | 6.372 | 0.928 | 1.452 | 0.603 | 0.016 |

| Contrast: AP_AF | | |  |  |  |  |  |
| --- | --- | --- | --- | --- | --- | --- | --- |
|  | average | sd | ratio | ava | avb | cumsum | P |
| ASA | 0.0151 | 0.0053 | 2.830 | 1.985 | 1.269 | 0.090 | 0.382 |
| MAN | 0.0117 | 0.0043 | 2.734 | 0.887 | 1.440 | 0.160 | 0.01 |
| GLC | 0.0110 | 0.0087 | 1.258 | 1.824 | 1.499 | 0.226 | 0.514 |
| INO | 0.0110 | 0.0019 | 5.735 | 0.567 | 1.086 | 0.291 | 0.001 |
| MAL | 0.0107 | 0.0041 | 2.646 | 0.910 | 1.202 | 0.355 | 0.017 |
| ARA | 0.0095 | 0.0042 | 2.251 | 0.948 | 1.399 | 0.412 | 0.91 |
| ALA | 0.0094 | 0.0036 | 2.625 | 1.229 | 0.783 | 0.469 | 0.744 |
| TRE | 0.0091 | 0.0063 | 1.438 | 1.355 | 1.058 | 0.523 | 0.154 |
| DHB | 0.0084 | 0.0031 | 2.722 | 0.848 | 0.452 | 0.573 | 0.003 |
| FRU | 0.0082 | 0.0049 | 1.667 | 1.625 | 1.695 | 0.622 | 0.5 |

| Contrast: AP_AA | | |  |  |  |  |  |
| --- | --- | --- | --- | --- | --- | --- | --- |
|  | average | sd | ratio | ava | avb | cumsum | P |
| ARA | 0.0270 | 0.0025 | 10.892 | 0.948 | 2.201 | 0.182 | 0.005 |
| ASA | 0.0201 | 0.0043 | 4.716 | 1.985 | 1.051 | 0.318 | 0.049 |
| ALA | 0.0127 | 0.0021 | 6.186 | 1.229 | 0.638 | 0.404 | 0.127 |
| TRE | 0.0083 | 0.0062 | 1.340 | 1.355 | 1.013 | 0.460 | 0.265 |
| GLN | 0.0067 | 0.0018 | 3.712 | 1.136 | 0.824 | 0.505 | 0.287 |
| LYS | 0.0066 | 0.0020 | 3.326 | 0.844 | 0.538 | 0.550 | 0.602 |
| MAT | 0.0063 | 0.0014 | 4.482 | 0.638 | 0.930 | 0.592 | 0.007 |
| GLC | 0.0060 | 0.0041 | 1.462 | 1.824 | 1.882 | 0.633 | 0.989 |

| Contrast: AF_AA | | |  |  |  |  |  |
| --- | --- | --- | --- | --- | --- | --- | --- |
|  | average | sd | ratio | ava | avb | cumsum | P |
| ARA | 0.0172 | 0.0040 | 4.351 | 1.399 | 2.201 | 0.125 | 0.175 |
| MAL | 0.0108 | 0.0039 | 2.772 | 1.202 | 0.929 | 0.203 | 0.016 |
| GLC | 0.0102 | 0.0088 | 1.163 | 1.499 | 1.882 | 0.277 | 0.693 |
| SUC | 0.0095 | 0.0062 | 1.542 | 1.294 | 0.851 | 0.346 | 0.014 |
| ASN | 0.0085 | 0.0038 | 2.243 | 1.360 | 0.966 | 0.408 | 0.007 |
| FRU | 0.0077 | 0.0051 | 1.500 | 1.695 | 1.641 | 0.464 | 0.556 |
| GLN | 0.0073 | 0.0061 | 1.186 | 1.163 | 0.824 | 0.517 | 0.175 |
| DHB | 0.0067 | 0.0019 | 3.589 | 0.452 | 0.766 | 0.566 | 0.047 |
| CIT | 0.0067 | 0.0019 | 3.569 | 0.892 | 0.580 | 0.614 | 0.02 |

average: average contribution of this species to the average dissimilarity between observations from the two groups. The sum of this column is the average dissimilarity between observations from the two groups; sd: standard deviation of the contribution of this substrates (i.e., based on its contribution to all dissimilarities between observations from the two groups); ratio: ratio of average to sd. Basically, a coefficient of variation (CV); ava, avb: average abundance of this substrate responses in each of the two groups; cumsum: cumulative contribution of this and all previous substrate responses in list, maximum value of this column is 1; P: permutation-based p-value; probability of getting a larger or equal average contribution for each substrate responses if the grouping factor was randomly permuted.

**Table S3.** Analysis of variance of the genus-based ngs data, model based on dbRDA reduced model with 5 factors, with permutation.

|  |  | Type I test |  |  | Type III test |  |  |
| --- | --- | --- | --- | --- | --- | --- | --- |
|  | Df | Sum of Sqs | F | p | Sum of Sqs | F | p |
| Model | 5 | 2.0388 | 5.94 | 0.001 |  |  | 0.001 |
| pH_KCl_ | 1 | 1.2608 | 18.37 | 0.001 | 0.5646 | 8.23 | 0.001 |
| NO_3_^–^-N | 1 | 0.2676 | 3.90 | 0.007 | 0.1597 | 2.33 | 0.047 |
| silt% | 1 | 0.1766 | 2.57 | 0.049 | 0.2245 | 3.27 | 0.014 |
| Mn | 1 | 0.1735 | 2.53 | 0.056 | 0.2303 | 3.36 | 0.016 |
| CaCO_3_ | 1 | 0.1603 | 2.34 | 0.065 | 0.1603 | 2.34 | 0.057 |
| Residual | 12 | 0.8235 |  |  | 0.8235 |  |  |

Analyses all terms separately in a sequential (Type I) test, terms added sequentially (first to last). Type III tests analyse the marginal effects when each term is eliminated from the model containing all other terms.
